# Association between Low Muscle Mass and Inflammatory Cytokines

**DOI:** 10.1155/2021/5572742

**Published:** 2021-04-27

**Authors:** Sadayuki Ito, Hiroaki Nakashima, Kei Ando, Kazuyoshi Kobayashi, Masaaki Machino, Taisuke Seki, Shinya Ishizuka, Ryosuke Fujii, Yasuhiko Takegami, Hiroya Yamada, Yoshitaka Ando, Koji Suzuki, Yukiharu Hasegawa, Shiro Imagama

**Affiliations:** ^1^Department of Orthopaedic Surgery, Nagoya University Graduate School of Medicine, Nagoya, Aichi, Japan; ^2^Department of Preventive Medical Sciences, Fujita Health University School of Medical Sciences, Aichi, Japan; ^3^Department of Hygiene, Fujita Health University School of Medicine, Aichi, Japan; ^4^Department of Biomedical and Analytical Sciences, Fujita Health University School of Medical Sciences, Aichi, Japan; ^5^Department of Rehabilitation, Kansai University of Welfare Science, Osaka, Japan

## Abstract

Sarcopenia is a multifaceted geriatric syndrome associated with the loss of muscle mass. We examined the relationship between low muscle mass and inflammatory cytokines in the context of aging. This study involved 299 participants (127 men and 172 women; mean age 63.3 ± 9.8 years) who underwent health checkups for body composition and inflammatory cytokine (TNF-alpha, IL-6, and MCP-1) levels. Muscle mass was determined using the skeletal muscle mass index. We divided the participants into the normal (N) and low muscle mass (L) groups and compared the levels of inflammatory cytokines in nonelderly (<65 years) and elderly (≥65 years) participants. Among the nonelderly subjects, C-reactive protein was significantly lower in the L group than in the N group (*p* < 0.05). However, there was no significant difference in the inflammatory cytokine levels between the groups. Among the elderly subjects, the TNF-alpha level was significantly lower in the L group than in the N group (*p* < 0.05), whereas there were no significant differences in the IL-6 and MCP-1 levels. Moreover, TNF-alpha was identified as a risk factor for the L group in the logistic regression analysis (Exp (B) 0.935, 95% CI: 0.876–0.997, *p* = 0.04). Although a low TNF-alpha level is a risk factor for low muscle mass, inflammatory cytokine levels are not necessarily elevated in elderly individuals with the loss of muscle mass.

## 1. Introduction

Age-related muscle atrophy, called sarcopenia, is the most common type of muscle atrophy in humans and is associated with significant functional impairments, such as slowing of movement and muscle weakness [1]. In recent years, the elderly population has increased owing to improvements in living conditions and health care. This has led to an increase in the proportion of elderly individuals with sarcopenia-related movement disorders and dependency problems. As a result, the socioeconomic consequences of sarcopenia have been increasing substantially, thereby increasing the importance of sarcopenia [2].

Sarcopenia is a multifaceted geriatric syndrome [3]. Sarcopenia is caused by the loss of nerve growth factors, growth hormones, androgens, estrogens, and other anabolic factors, along with decreased physical activity, anorexia, increased catabolic factors such as inflammatory cytokines, decreased muscle fiber number, and increased abnormal muscle firing.

Chronic inflammation-induced imbalances in protein synthesis and metabolism have been shown to cause muscle loss [4, 5]. Thus, there have been several studies on the relationship between muscle and inflammatory markers [4–7]; among the inflammatory markers, inflammatory cytokines are noteworthy. While some studies have shown inflammatory cytokines to be elevated in sarcopenia [6, 7], the results of other studies are contradictory. In this study, we analyzed inflammatory cytokines as a cause of muscle loss.

Most studies on the relationship between inflammatory cytokines and muscle mass have focused on cases of chronic inflammation, such as chronic diseases and obesity, and cases of impaired functions in addition to the loss of muscle mass [8]. Therefore, the relationship between inflammatory cytokines and mild and early-onset sarcopenia has not been fully understood. Studies have shown that muscle plasticity actually decreases with age [9]. Therefore, early detection and intervention for muscle loss may help prevent sarcopenia. Identifying where muscle loss starts in the body may enable the early diagnosis of sarcopenia.

The aim of this study was to investigate the relationship between muscle mass and inflammatory cytokines using data from healthy humans to determine factors contributing to muscle loss.

## 2. Materials and Methods

### 2.1. Participants

Participants in this study were volunteers who underwent health checkups supported by the town of Yakumo, Japan, in 2015. Yakumo Town has a population of approximately 17,000 people, of which 28% are over 65 years old. More people in Yakumo Town are engaged in agriculture and fishery than those in urban areas. Since 1982, resident checkups have been conducted annually in this town. The checkup consists of orthopedic and physical function examinations, internal medical examinations, and psychological tests, as well as a health-related quality of life survey [10].

Bioelectrical impedance analysis (BIA) was used to analyze the body composition of the participants. The participants underwent the BIA on an empty stomach. The conditions of BIA measurement, such as consumption of food and beverages, were similar to those reported earlier [11].

We included participants who underwent both BIA measurement and blood tests. The exclusion criteria were as follows: history of spine and limb joint surgery; severe knee injury; severe osteoarthritis; history of fracture in the hip and spine; neurological disorders; severe mental illness; kidney or heart disease; chronic inflammatory diseases, including rheumatoid arthritis; consumption of food; severe disability in walking or standing; or any dysfunction of the central or peripheral nervous systems. Among 525 individuals who underwent health checkups, 299 participants (127 men and 172 women) met the inclusion criteria.

The study protocol was approved by the ethics committee of the institutional review board of our university. All participants provided written informed consent before participation. The study procedures were carried out in accordance with the principles of the Declaration of Helsinki.

### 2.2. Data Measurement

Anthropometric data, including height, weight, body mass index (BMI), and each limb's skeletal muscle mass index (SMI), were measured using the BIA. The Inbody 770 BIA device (Inbody Co., Ltd., Seoul, Korea), which can differentiate tissues (such as fat, muscle, and bone) based on their electrical impedance, was used for the purpose [12]. The accuracy of this device has been reported previously [13, 14]. Participants grasped the handles of the analyzer, which have embedded electrodes, and stood on the platform with the soles of the feet in contact with the electrodes. There were two electrodes for each foot and hand. The BMI was calculated using the following formula: weight (kg)/height^2^ (m^2^). Muscle mass of each limb was automatically calculated by BIA using the Inbody 770 BIA device. The SMI of each limb was calculated using the following formula: each limb′s SMI = each limb′s skeletal muscle mass (kg)/height^2^ (m^2^) [15].

Low muscle mass was defined as having an appendicular SMI (ASMI/height^2^) of less than 7.0 kg/m^2^ and 5.7 kg/m^2^ in men and women, respectively, according to the Asian Working Group for Sarcopenia criteria [16]. We divided the participants into the normal muscle mass (N) group (ASMI ≥ 7.0 kg/m^2^ and 5.7 kg/m^2^ in men and women, respectively) and the lower muscle mass (L) group (ASMI < 7.0 kg/m^2^ and 5.7 kg/m^2^ in men and women, respectively).

During the checkup, fasting blood samples were collected by venipuncture and centrifuged within 1 h of sampling. The serum samples were stored at −80°C until further analysis. Routine biochemical analyses were performed in the laboratory at Yakumo Town Hospital.

A custom-made, human premixed multianalyte kit (Magnetic Luminex Screening Assay, R&D Systems, Minneapolis, MN) was used to measure TNF-alpha, IL-6, and MCP-1 [17].

### 2.3. Statistical Analysis

Continuous variables are expressed as the mean ± standard deviation. We compared the continuous variables of the L group with those of the N group using the Mann–Whitney *U*-test. We also compared the categorical variables of the L group with those of the N group using the chi-squared test. Spearman's rank correlation coefficient was used to examine the relationship between the SMI measured by BIA and TNF-alpha level. A logistic regression analysis was used to examine the risk factors for low muscle mass and was performed for items that were significantly different (*p* < 0.05) in the univariate analysis. Each analysis was performed separately for the under-65 group (nonelderly) and the over-65 group (elderly).

All statistical analyses were performed using SPSS Statistics v.22.0 software for Mac (IBM Corp., Armonk, NY, USA). Results with a *p* value of <0.05 were considered significant in all analyses.

## 3. Results

Participant characteristics are shown in Table 1. There were 127 men and 172 women, with an average age of 63.3 ± 9.8 years. The mean SMI was 6.96 ± 1.05 kg/m^2^. There were 257 and 42 participants in the N and L groups, respectively. The mean levels of inflammatory cytokines were as follows: TNF-alpha, 11.3 ± 10.9 pg/ml; IL-6, 7.9 ± 26.8 pg/ml; and MCP-1, 61.3 ± 59.6 pg/ml.

### 3.1. Nonelderly Participants

In the nonelderly group, the average age of the participants was 55.5 ± 7.1 years, and the mean SMI was 6.9 ± 1.1 kg/m^2^. In total, 127 (87.6%) and 18 (12.4%) subjects were included in the N and L groups, respectively. The mean TNF-alpha, IL-6, and MCP-1 levels were 11.5 ± 10.70, 9.61 ± 37.37, and 62.7 ± 54.60 pg/ml, respectively (Table 1). The average age of participants was significantly higher in the L group than in the N group (N: 55.1 ± 7.2, L: 58.1 ± 5.0, *p* = 0.004). The BMI and body fat percentage (BFP) were significantly lower in the L group than in the N group (BMI: N: 24.0 ± 3.4, L: 19.5 ± 2.0, *p* < 0.001; BFP: N: 29.9 ± 7.6, L: 25.9 ± 5.7, *p* = 0.02). In terms of the laboratory data and inflammatory cytokines, C-reactive protein (CRP) was significantly lower in the L group than in the N group (N: 0.06 ± 0.11, L: 0.03 ± 0.05, *p* = 0.046). There were no significant differences in the other laboratory data, including inflammatory cytokines, gait speed, and history of smoking or metabolic diseases, between the N and L groups (Table 2). In both the nonelderly female and male groups, there were no significant differences in inflammatory cytokines between the N and L groups (Supplemental Tables 1 and 2).

### 3.2. Elderly Participants

The average age of the participants was 70.7 ± 5.2 years, and the mean SMI was 7.0 ± 1.0 kg/m^2^. The N and L groups contained 130 (84.4%) and 24 (15.6%) elderly subjects, respectively. The mean TNF-alpha, IL-6, and MCP-1/CCL2 levels were 11.0 ± 11.2, 6.9 ± 8.9, and 59.9 ± 64.0 pg/ml, respectively (Table 1).

**Figure 1:**
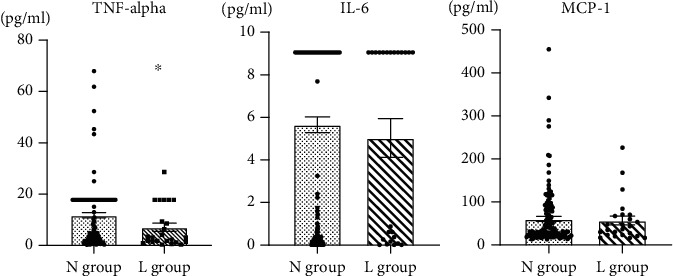
Comparison of inflammatory cytokines between the N and L groups. TNF-alpha of the L group is significantly lower than that of the N group. There is no significant difference in IL-6 and MCP-1 between the N and L groups. ^∗^*p* < 0.05.

The average age of participants was significantly higher in the L group than in the N group (N: 74.20 ± 6.65, L: 70.0 ± 4.7, *p* = 0.002). The BMI was significantly lower in the L group than in the N group (N: 24.1 ± 3.1, L: 20.9 ± 2.5, *p* < 0.001). In terms of the laboratory data and inflammatory cytokines, only TNF-alpha was significantly lower in the L group than in the N group (N: 11.8 ± 11.6, L: 7.1 ± 8.0, *p* = 0.041). There were no significant differences in the other laboratory data, including inflammatory cytokines, gait speed, and history of smoking or metabolic diseases, between the N and L groups (Table 3, Figure 1). In the nonelderly female group, only TNF-alpha of the inflammatory cytokines was significantly lower in the L group than in the N group (N: 11.15 ± 11.04, L: 5.68 ± 7.06, *p* = 0.031). There were no significant differences in the other inflammatory cytokines between the N and L groups (Supplemental Table 3). In the nonelderly male group, there were no significant differences in inflammatory cytokines between the N and L groups (Supplemental Table 4).

As significant differences were observed among several factors, they were examined as covariates for risk factors for the L group in the logistic regression analysis. The results indicated age, BMI, and TNF-alpha as risk factors (age: Exp (B) 1.185, 95% CI: 1.073–1.308, *p* = 0.001; BMI: Exp (B) 0.599, 95% CI: 0.471–0.761, *p* < 0.001; and TNF-alpha: Exp (B) 0.935, 95% CI: 0.876–0.997, *p* = 0.04) (Table 4). In addition, we found a positive correlation between SMI and TNF-alpha (*r* = 0.159, *p* = 0.049).

## 4. Discussion

In the present study, we found that the CRP level was lower in the L group than in the N group among the nonelderly subjects, and the TNF-alpha level was lower in the L group than in the N group among the elderly individuals. This indicates that inflammatory markers were reduced in the low muscle mass group.

Inflammation is considered a cause of sarcopenia; however, most available information is about the role of inflammation and inflammatory cytokines in acute disease. In a typical acute immune response, antigen-presenting cells that encounter foreign peptides secrete IL-1 and TNF-alpha, which help the recruitment of T cells and development of specific immune responses to antigens [18]. IL-1 and TNF-alpha are endogenous pyrogens and have significant effects on metabolism in acute disease, including altered secretion of insulin and insulin antagonist hormones (glucagon, epinephrine, and cortisol), increased production of glucose, increased proteolysis, and increased production of liver glucose. Furthermore, these cytokines, along with IL-6, upregulate CRP and other positive acute phase reactants and downregulate albumin gene transcription, thereby causing an acute phase response.

Inflammatory cytokines are elevated in elderly individuals, although their levels are well below those in acute infections [19]. Increased levels of TNF-alpha, IL-1, and IL-6 activate the ubiquitin-proteasome proteolytic pathway [8, 20] and play important roles in low-grade systemic inflammation; moreover, they have been implicated in the development of sarcopenia [21]. Although the mechanisms underlying age-related low-grade inflammation are not yet fully understood, various factors are known to contribute to it. These include immune responses, a sedentary lifestyle, pathological conditions such as increased subcutaneous adipose tissue, the presence of undetected infections, chronic health problems, and nutritional changes. However, there was no increase in the TNF or IL-6 level in the group with reduced muscle mass in the present study.

TNF-alpha is a representative regulator of the apoptotic signaling pathway. It binds to the TNF-alpha receptor in skeletal muscle and activates caspases via the Fas-associated death domain to induce apoptosis [22]. Excessive upregulation of apoptosis in skeletal muscle increases the breakdown of muscle proteins and muscle atrophy. However, in the present study, the TNF-alpha level was reduced in the low muscle mass group. TNF acts on adipose tissue, besides skeletal muscle, increasing free fatty acids and causing chronic inflammation, which may have affected the results of this study.

IL-6 is not only an inflammatory cytokine but also a myokine. It plays a role in promoting the utilization of fat in adipose tissue and the liver. In skeletal muscle, IL-6 plays a role in promoting the utilization of intramuscular fat. IL-6 also exhibits inflammatory and anti-inflammatory effects and acts in an anti-inflammatory manner when secreted during muscle contraction [23]. However, it is not possible to determine whether IL-6 secreted into the blood is of muscle origin. Thus, owing to its both inflammatory and anti-inflammatory effects, the role of IL-6 is still controversial in sarcopenia. In the present study, we found no significant association between IL-6 and sarcopenia.

MCP-1 is considered to be involved in the tissue infiltration of monocytes and T cells in various inflammatory diseases [24, 25]. In skeletal muscle, MCP-1 is a chemokine produced during muscle injury and acts on the chemokine receptor CCR2 on bone marrow-derived monocytes, causing macrophage accumulation in the site of injury and inflammation. CCL2-induced macrophages produce IGF-1, which is necessary for myoblast proliferation. It is known that CCL2-deficient mice do not produce IGF-1 locally, which delays the recovery of injured muscle [26]. Although MCP-1 is elevated in inflammation, it also has the necessary proliferative effect on muscle. In this study, MCP-1 was not found to be associated with sarcopenia.

Although some studies have shown that proinflammatory cytokines are elevated in sarcopenia, other studies including the present study did not find such elevated levels [4–7]. Sarcopenia is thought to develop slowly over 10 years, with subtle changes in the balance between muscle protein catabolism and anabolism resulting in major changes in body composition and the development of sarcopenia. Although we hypothesized the elevation of cytokines in sarcopenia based on the theoretical possibility that the elevation in proinflammatory cytokines may promote protein catabolism, our results did not support this hypothesis. Thus, it is highly likely that sarcopenia is different from the rapid histological changes mediated by cytokines observed in acute inflammation. The present study results may have important implications for the early pathology of sarcopenia.

This study had a few potential limitations. First, the participants were middle-aged and elderly people who lived in a relatively rural area where many are involved in agriculture or fishing as their occupation. Therefore, the subjects differed from people in an urban environment, and the results of this study may not be directly applied to such a population. Second, this was a cross-sectional, single-center study. Further longitudinal and multicenter collaborative studies are needed to verify our findings.

## 5. Conclusions

In conclusion, this study showed that TNF-alpha was lower in elderly individuals showing the loss of muscle mass than in healthy human subjects. In addition, IL-6 and MCP-1 were not associated with the loss of muscle mass. This study suggests that inflammatory cytokines are not necessarily elevated in elderly individuals with the loss of muscle mass.

## Figures and Tables

**Table 1:** Demographics, clinical characteristics, laboratory data, and inflammatory cytokines.

	Total (*n* = 299)	Nonelderly (*n* = 145)	Elderly (*n* = 154)	*p*
Male/female	127/172	49/96	78/76	0.004
Age (yrs)	63.3 ± 9.8	55.5 ± 7.1	70.7 ± 5.2	<0.001
BMI (kg/m^2^)	23.5 ± 3.4	23.4 ± 3.6	23.6 ± 3.2	0.616
ASMI (kg/m^2^)	6.96 ± 1.05	6.94 ± 1.12	6.97 ± 0.98	0.806
N/L	257/42	127/18	130/24	0.506
BFP (%)	28.4 ± 7.1	29.4 ± 7.5	27.4 ± 6.6	0.014
Smoking history (y/n)	39/260	22/123	17/137	0.307
Hypertension (y/n)	90/209	24/121	66/88	<0.001
Diabetes (y/n)	22/277	9/136	13/141	0.303
Hyperlipidemia (y/n)	92/207	33/112	59/95	0.003
HbA1c (%)	5.7 ± 0.5	5.6 ± 0.4	5.8 ± 0.5	<0.001
Alb (serum albumin) (g/dl)	4.29 ± 0.26	4.32 ± 0.25	4.26 ± 0.26	<0.049
AST (aspartate transaminase) (U/I)	23.1 ± 8.0	21.4 ± 7.1	24.8 ± 8.5	<0.001
ALT (alanine aminotransferase) (U/I)	22.6 ± 12.7	21.86 ± 13.06	23.2 ± 12.4	0.355
T-cho (mg/dl)	211.7 ± 35.7	216.8 ± 34.98	206.8 ± 35.9	0.015
BUN (mg/dl)	15.2 ± 4.0	13.92 ± 3.28	16.38 ± 4.28	<0.001
Cre (mg/dl)	0.77 ± 0.27	0.71 ± 0.13	0.82 ± 0.34	<0.001
CRP (mg/dl)	0.09 ± 0.27	0.06 ± 0.1	0.12 ± 0.36	0.039
TNF-alpha (pg/ml)	11.3 ± 11.0	11.5 ± 10.7	11.0 ± 11.2	0.695
IL-6 (pg/ml)	7.9 ± 26.8	9.6 ± 37.4	6.2 ± 8.9	0.270
MCP-1 (pg/ml)	61.3 ± 59.6	62.7 ± 54.6	59.9 ± 64.1	0.688

BMI: body mass index; BFP: body fat percentage; ASMI: appendicular skeletal muscle mass; appendicular soft lean mass/(height)^2^; N/L: N group (ASMI ≥ 7.0 kg/m^2^ and 5.7 kg/m^2^ in men and women, respectively)/L group (ASMI < 7.0 kg/m^2^ and 5.7 kg/m^2^ in men and women, respectively).

**Table 2:** Comparison of demographics, clinical characteristics, laboratory data, and inflammatory cytokines between the N and L groups in nonelderly participants.

Nonelderly	N (*n* = 127)	L (*n* = 18)	*p*
Male/female	45/82	4/14	0.202
Age (yrs)	55.1 ± 7.2	58.1 ± 5.0	0.004
BMI (kg/m^2^)	24.0 ± 3.4	19.5 ± 2.0	<0.001
BFP (%)	29.9 ± 7.6	25.9 ± 5.7	0.017
Gait speed (m/s)	2.67 ± 0.42	2.60 ± 0.40	0.502
Smoking history (y/n)	20/107	2/16	1.000
Hypertension (y/n)	22/105	2/16	0.394
Diabetes (y/n)	8/119	1/17	0.690
Hyperlipidemia (y/n)	31/96	2/16	0.170
HbA1c (%)	5.6 ± 0.4	5.5 ± 0.5	0.276
Alb (g/dl)	4.32 ± 0.24	4.27 ± 0.32	0.472
AST (U/I)	21.2 ± 6.3	22.5 ± 11.3	0.833
ALT (U/I)	22.2 ± 13.2	19.8 ± 12.0	0.248
T-cho (mg/dl)	217.4 ± 36.0	212.8 ± 27.0	0.869
BUN (mg/dl)	13.8 ± 3.3	14.6 ± 3.5	0.393
Cre (mg/dl)	0.71 ± 0.13	0.66 ± 0.12	0.129
CRP (mg/dl)	0.06 ± 0.11	0.03 ± 0.05	0.046
TNF-alpha (pg/ml)	11.7 ± 11.0	10.2 ± 8.2	0.776
IL-6 (pg/ml)	10.2 ± 39.9	5.8 ± 4.3	0.823
MCP-1 (pg/ml)	61.3 ± 53.7	72.7 ± 60.7	0.328

BMI: body mass index; BFP: body fat percentage; N: N group (ASMI ≥ 7.0 kg/m^2^ and 5.7 kg/m^2^ in men and women, respectively); L: L group (ASMI < 7.0 kg/m^2^ and 5.7 kg/m^2^ in men and women, respectively); ASMI: appendicular skeletal muscle mass; appendicular soft lean mass/(height)^2^.

**Table 3:** Comparison of demographics, clinical characteristics, laboratory data, and inflammatory cytokines between the N and L groups in elderly participants.

Elderly	N (*n* = 130)	L (*n* = 24)	*p*
Male/female	67/63	11/13	0.385
Age (yrs)	70.0 ± 4.7	74.2 ± 6.7	0.002
BMI (kg/m^2^)	24.1 ± 3.1	20.9 ± 2.5	<0.001
BFP (%)	27.7 ± 6.6	25.5 ± 6.2	0.082
Gait speed (m/s)	2.63 ± 0.46	2.75 ± 0.46	0.257
Smoking history (y/n)	13/117	4/20	0.307
Hypertension (y/n)	57/73	9/15	0.365
Diabetes (y/n)	9/121	4/20	0.122
Hyperlipidemia (y/n)	50/80	9/15	0.560
HbA1c (%)	5.8 ± 0.5	5.8 ± 0.5	0.889
Alb (g/dl)	4.26 ± 0.27	4.22 ± 0.16	0.323
AST (U/I)	24.4 ± 8.0	27.0 ± 11.0	0.356
ALT (U/I)	23.9 ± 13.0	19.8 ± 8.0	0.185
T-cho (mg/dl)	207.2 ± 34.8	204.9 ± 41.9	0.464
BUN (mg/dl)	16.4 ± 4.3	16.2 ± 4.2	0.936
Cre (mg/dl)	0.83 ± 0.37	0.78 ± 0.10	0.974
CRP (mg/dl)	0.12 ± 0.36	0.15 ± 0.34	0.707
TNF-alpha (pg/ml)	11.8 ± 11.6	7.0 ± 8.0	0.041
IL-6 (pg/ml)	6.4 ± 9.5	5.0 ± 4.5	0.442
MCP-1 (pg/ml)	60.5 ± 66.3	56.7 ± 51.5	0.864

BMI: body mass index. BFP: body fat percentage; N: N group (ASMI ≥ 7.0 kg/m^2^ and 5.7 kg/m^2^ in men and women, respectively); L: L group (ASMI < 7.0 kg/m^2^ and 5.7 kg/m^2^ in men and women, respectively); ASMI: appendicular skeletal muscle mass; appendicular soft lean mass/(height)^2^.

**Table 4:** Logistic regression analysis for risk factors for the L group in elderly participants.

Elderly	*B*	SE	Wald	df	*p*	Exp (B)	95% CI
Age	0.17	0.051	11.287	1	0.001	1.185	1.073-1.308
Sex	0.956	0.611	2.451	1	0.117	2.602	0.786-8.618
BMI	-0.512	0.122	17.526	1	<0.001	0.599	0.471-0.761
TNF-alpha	-0.068	0.033	4.227	1	0.04	0.935	0.876-0.997

L: L group (ASMI < 7.0 kg/m^2^ and 5.7 kg/m^2^ in men and women, respectively); BMI: body mass index.

## Data Availability

The data of health checkups used to support the findings of this study are available from the corresponding author upon request.

## Supplementary Materials

Supplementary Materials Supplemental Table 1: comparison of demographics, clinical characteristics, laboratory data, and inflammatory cytokines between the N and L groups in nonelderly female participants. Supplemental Table 2: comparison of demographics, clinical characteristics, laboratory data, and inflammatory cytokines between the N and L groups in nonelderly male participants. Supplemental Table 3: comparison of demographics, clinical characteristics, laboratory data, and inflammatory cytokines between the N and L groups in elderly female participants. Supplemental Table 4: comparison of demographics, clinical characteristics, laboratory data, and inflammatory cytokines between the N and L groups in elderly male participants.
