# Supplementary material for: Association between Low Muscle Mass and Inflammatory Cytokines
Source: Biomed Res Int. 2021 Apr 27;2021:5572742. doi: 10.1155/2021/5572742 (PMC8099521; doi:10.1155/2021/5572742)
Supplement: Supplementary Materials — Supplemental Table 1: comparison of demographics, clinical characteristics, laboratory data, and inflammatory cytokines between the N and L groups in nonelderly female participants. Supplemental Table 2: comparison of demographics, clinical characteristics, laboratory data, and inflammatory cytokines between the N and L groups in nonelderly male participants. Supplemental Table 3: comparison of demographics, clinical characteristics, laboratory data, and inflammatory cytokines between the N and L groups in elderly female participants. Supplemental Table 4: comparison of demographics, clinical characteristics, laboratory data, and inflammatory cytokines between the N and L groups in elderly male participants. [file 5572742.f1.docx]

**Supplemental Table1:** Comparison of Demographics, clinical characteristics, laboratory data and inflammatory cytokines between N and L group in non-elderly female participants

| non-elderly female | N(n=82) | L(n=14) | p |
| --- | --- | --- | --- |
| age(yrs) | 54.57±7.5 | 57.35±5.19 | 0.098 |
| BMI(kg/m2) | 23.57±3.57 | 19.28±1.99 | <0.001 |
| BFP(%) | 33.51±6.24 | 27.4±5.31 | <0.001 |
| Gait speed(m/s) | 2.67±0.41 | 2.54±0.42 | 0.380 |
| Smoking history(y/n) | 9/73 | 1/13 | 0.533 |
| Hypertension(y/n) | 14/68 | 2/12 | 0.576 |
| Diabetes(y/n) | 5/77 | 1/13 | 0.622 |
| Hyperlipidemia(y/n) | 20/62 | 2/12 | 0.327 |
| HbA1c(%) | 5.56±0.37 | 5.43±0.49 | 0.376 |
| Alb(g/dl) | 4.32±0.23 | 4.29±0.34 | 0.736 |
| AST(U/I) | 19.57±4.79 | 20.57±5.4 | 0.525 |
| ALT(U/I) | 18.47±9.05 | 17.64±6.97 | 0.698 |
| T-cho(mg/dl) | 219.46±40.09 | 212.78±28.22 | 0.453 |
| BUN(mg/dl) | 13.23±3.19 | 13.94±3.06 | 0.435 |
| Cre(mg/dl) | 0.64±0.09 | 0.62±0.09 | 0.455 |
| CRP(mg/dl) | 0.04±0.06 | 0.03±0.06 | 0.512 |
| TNF-alpha(pg/ml) | 11.87±11.09 | 10.08±8.87 | 0.512 |
| IL6(pg/ml) | 6.27±4.24 | 6.71±3.89 | 0.703 |
| MCP-1(pg/ml) | 59.48±54.55 | 65.96±50.96 | 0.668 |

BMI: Body Mass Index

BFP: Body Fat Percentage

N: N group (ASMI≧7.0 kg/m^2^ and 5.7 kg/m^2^ in men and women respectively)

L: L group (ASMI<7.0 kg/m^2^ and 5.7 kg/m^2^ in men and women respectively)

ASMI: appendicular skeletal muscle mass; appendicular soft lean mass/(height)2

**Supplemental Table2:** Comparison of Demographics, clinical characteristics, laboratory data and inflammatory cytokines between N and L group in non-elderly male participants

| non-elderly male | N(n=45) | L(n=4) | p |
| --- | --- | --- | --- |
| age(yrs) | 55.66±6.49 | 60.5±3.87 | 0.08 |
| BMI(kg/m2) | 24.7±2.97 | 20.37±1.88 | 0.011 |
| BFP(%) | 23.34±5.01 | 20.8±3.67 | 0.268 |
| Gait speed(m/s) | 2.68±0.44 | 2.74±0.32 | 0.744 |
| Smoking history(y/n) | 11/34 | 1/3 | 0.668 |
| Hypertension(y/n) | 8/37 | 0/4 | 0.478 |
| Diabetes(y/n) | 3/42 | 0/4 | 0.77 |
| Hyperlipidemia(y/n) | 11/34 | 0/4 | 0.348 |
| HbA1c(%) | 5.69±0.43 | 5.75±0.7 | 0.883 |
| Alb(g/dl) | 4.33±0.26 | 4.2±0.29 | 0.445 |
| AST(U/I) | 24.28±7.55 | 29.25±22.77 | 0.693 |
| ALT(U/I) | 28.86±16.68 | 27.5±22.54 | 0.913 |
| T-cho(mg/dl) | 213.55±27.09 | 213±25.96 | 0.97 |
| BUN(mg/dl) | 14.9±3.12 | 17.07±4.11 | 0.372 |
| Cre(mg/dl) | 0.84±0.12 | 0.8±0.11 | 0.53 |
| CRP(mg/dl) | 0.1±0.16 | 0.03±0.03 | 0.512 |
| TNF-alpha(pg/ml) | 11.44±10.86 | 10.81±6.16 | 0.396 |
| IL6(pg/ml) | 17.23±66.66 | 2.55±4.33 | 0.155 |
| MCP-1(pg/ml) | 64.53±52.67 | 96.24±93.31 | 0.548 |

BMI: Body Mass Index

BFP: Body Fat Percentage

N: N group (ASMI≧7.0 kg/m^2^ and 5.7 kg/m^2^ in men and women respectively)

L: L group (ASMI<7.0 kg/m^2^ and 5.7 kg/m^2^ in men and women respectively)

ASMI: appendicular skeletal muscle mass; appendicular soft lean mass/(height)2

**Supplemental Table3:** Comparison of Demographics, clinical characteristics, laboratory data and inflammatory cytokines between N and L group in elderly female participants

| elderly female | N(n=63) | L(n=13) | p |
| --- | --- | --- | --- |
| age(yrs) | 70.03±4.5 | 76±6.44 | 0.06 |
| BMI(kg/m2) | 23.61±3.66 | 20.32±3.03 | 0.003 |
| BFP(%) | 31.79±6.34 | 28.19±6.7 | 0.093 |
| Gait speed(m/s) | 2.57±0.50 | 2.62±0.44 | 0.769 |
| Smoking history(y/n) | 2/61 | 1/12 | 0.435 |
| Hypertension(y/n) | 23/40 | 4/9 | 0.478 |
| Diabetes(y/n) | 2/61 | 4/9 | 0.007 |
| Hyperlipidemia(y/n) | 26/37 | 7/6 | 0.298 |
| HbA1c(%) | 5.73±0.43 | 5.99±0.64 | 0.195 |
| Alb(g/dl) | 4.26±0.23 | 4.23±0.14 | 0.578 |
| AST(U/I) | 24.09±8.72 | 26.61±9.02 | 0.369 |
| ALT(U/I) | 22.06±11.87 | 19.38±7.47 | 0.304 |
| T-cho(mg/dl) | 216.15±30.84 | 213.23±40.66 | 0.809 |
| BUN(mg/dl) | 15.45±3 | 17.87±4.45 | 0.082 |
| Cre(mg/dl) | 0.69±0.1 | 0.77±0.12 | 0.043 |
| CRP(mg/dl) | 0.08±0.14 | 0.18±0.46 | 0.435 |
| TNF-alpha(pg/ml) | 11.15±11.04 | 5.68±7.06 | 0.031 |
| IL6(pg/ml) | 6.4±4.03 | 6.33±4.25 | 0.955 |
| MCP-1(pg/ml) | 71.38±78.26 | 58.4±47.18 | 0.435 |

BMI: Body Mass Index

BFP: Body Fat Percentage

N: N group (ASMI≧7.0 kg/m^2^ and 5.7 kg/m^2^ in men and women respectively)

L: L group (ASMI<7.0 kg/m^2^ and 5.7 kg/m^2^ in men and women respectively)

ASMI: appendicular skeletal muscle mass; appendicular soft lean mass/(height)2

**Supplemental Table4:** Comparison of Demographics, clinical characteristics, laboratory data and inflammatory cytokines between N and L group in elderly male participants

| elderly male | N(n=67) | L(n=11) | p |
| --- | --- | --- | --- |
| age(yrs) | 70.04±4.86 | 71.63±6.81 | 0.472 |
| BMI(kg/m2) | 24.59±2.33 | 21.64±1.64 | <0.001 |
| BFP(%) | 23.94±4.12 | 22.4±3.79 | 0.236 |
| Gait speed(m/s) | 2.67±0.43 | 2.91±0.46 | 0.155 |
| Smoking history(y/n) | 11/56 | 3/8 | 0.31 |
| Hypertension(y/n) | 34/33 | 5/6 | 0.5 |
| Diabetes(y/n) | 7/60 | 0/11 | 0.329 |
| Hyperlipidemia(y/n) | 24/43 | 2/9 | 0.214 |
| HbA1c(%) | 5.85±0.6 | 5.65±0.35 | 0.132 |
| Alb(g/dl) | 4.27±0.31 | 4.2±0.18 | 0.32 |
| AST(U/I) | 24.61±7.3 | 27.36±13.49 | 0.522 |
| ALT(U/I) | 25.58±13.8 | 20.18±8.93 | 0.106 |
| T-cho(mg/dl) | 198.73±36.42 | 195±42.99 | 0.79 |
| BUN(mg/dl) | 17.31±5.11 | 14.17±2.98 | 0.052 |
| Cre(mg/dl) | 0.96±0.47 | 0.79±0.09 | 0.241 |
| CRP(mg/dl) | 0.16±0.48 | 0.11±0.14 | 0.537 |
| TNF-alpha(pg/ml) | 12.35±12.17 | 8.69±9.07 | 0.255 |
| IL6(pg/ml) | 6.4±12.72 | 3.49±4.41 | 0.162 |
| MCP-1(pg/ml) | 50.27±51.14 | 54.73±58.4 | 0.815 |

BMI: Body Mass Index

BFP: Body Fat Percentage

N: N group (ASMI≧7.0 kg/m^2^ and 5.7 kg/m^2^ in men and women respectively)

L: L group (ASMI<7.0 kg/m^2^ and 5.7 kg/m^2^ in men and women respectively)

ASMI: appendicular skeletal muscle mass; appendicular soft lean mass/(height)2
